# Clinicopathological characteristics of synchronous multiple primary early esophageal cancer and risk factors for multiple lesions

**DOI:** 10.3389/fonc.2023.1219451

**Published:** 2023-08-17

**Authors:** Jing Su, Shuchun Wei, Wenjie Li, Han Chen, Lurong Li, Lijuan Xu, Ping Zhao, Guoxin Zhang, Jin Yan

**Affiliations:** ^1^ Department of Gastroenterology, The First Affiliated Hospital of Nanjing Medical University, Nanjing, Jiangsu, China; ^2^ Department of Gastroenterology, Xuzhou Central Hospital, The Xuzhou School of Clinical Medicine of Nanjing Medical University, Xuzhou, Jiangsu, China

**Keywords:** risk factors, main lesions, accessory lesions, endoscopic submucosal dissection (ESD), synchronous multiple primary early esophageal cancer

## Abstract

**Background:**

With the development of endoscopic technology, the detection rate of synchronous multiple primary early esophageal cancer (SMPEEC) is increasing; however, the risk factors remain unclear. We aimed to assess the clinicopathological characteristics of patients with SMPEEC and investigate the risk factors contributing to the development of multiple lesions.

**Methods:**

A retrospective cohort study was conducted on 911 consecutive patients who underwent Endoscopic submucosal dissection (ESD) for primary esophageal neoplasms from January 2013 to June 2021. The patients were divided into the SMPEEC group and the solitary early esophageal cancer (SEEC) group. We compared the differences in clinicopathological characteristics between the two groups and investigated the risk factors linked to multiple lesions. Additionally, we investigated the relationship between the main and accessory lesions.

**Results:**

A total of 87 SMPEEC patients were included in this study, and the frequency of synchronous multiple lesions was 9.55% in patients with early esophageal cancer. The lesions in the SMPEEC group were mainly located in the lower segment of the esophagus (46[52.9%]), whereas those in the SEEC group were in the middle segment (412[50.0%]). The pathology type, tumor location, and circumferential rate of lesions were independent risk factors(*P*<0.05) for SMPEEC by logistic regression analysis. Significant positive correlations were observed between the main and accessory lesions in terms of morphologic type (r=0.632, *P*=0.000), tumor location(r=0.325, *P*=0.037), pathologic type (r=0.299, *P*=0.003), and depth of invasion (r=0.562, *P*=0.000).

**Conclusion:**

Pathology type, tumor location, and circumferential rate of lesions were identified as independent risk factors for SMEPPC. Understanding these risk factors and the correlation between the main and accessory lesions could significantly improve the detection rate of SMPEEC.

## 1 Introduction

Synchronous multiple primary esophageal cancer (SMPEC) is a relatively rare and aggressive tumor, defined as two or more carcinomas in different parts of the esophagus confirmed by pathological examination either simultaneously or successively within 6 months (1, 2). The reported incidence of SMPEC varies from 0.1 to 10.0% (3, 4). The treatment of SMPEC is usually based on surgery or radiotherapy and chemotherapy, and the prognosis is poor, with a five-year survival rate of less than 30% (5).

With the development of endoscopic technology and increased awareness of early cancer screening, the detection rate of synchronous multiple primary early esophageal cancer (SMPEEC) is rising. Endoscopic submucosal dissection (ESD) has been widely used to treat early esophageal cancer, with a high curative resection rate and minimal trauma (6). However, in cases of multiple early esophageal cancer or precancerous lesions, small or flat lesions often remain undetected during endoscopic examinations, increasing the risk of progression to advanced cancer and depriving patients of the opportunity for curative resection. It has been reported that the underdiagnosis rate of esophageal high-grade intraepithelial neoplasia can be as high as 45% in high-risk patients with esophageal cancer (7). Thus, it is crucial to improve the detection rate of SMPEEC to optimize patient management and treatment strategies.

Limited reports exist regarding the treatment of SMPEEC with ESD. Previous studies have predominantly focused on advanced multiple esophageal carcinomas or solitary early esophageal cancer (SEEC), leaving SMPEEC relatively unexplored. Hence, elucidating the clinical and pathological characteristics of SMPEEC holds significant importance in enabling accurate clinical diagnosis and effective treatment strategies. This study aimed to provide a comprehensive summary of the characteristics of SMPEEC and investigate the risk factors linked to multiple lesions. The efficacy, recurrence rate, and safety of ESD for treating multiple esophageal lesions were also evaluated. Additionally, we explored the relationship between the main and accessory lesions of SMPEEC.

## 2 Methods

### 2.1 Patients

Patients who underwent esophageal ESD between January 2013 and June 2021 at The First Affiliated Hospital of Nanjing Medical University (Nanjing, China) were retrospectively analyzed. This study was conducted in accordance with the Declaration of Helsinki and was approved by the Institutional Review Board of The First Affiliated Hospital of Nanjing Medical University (No. 2018-SR-272).

### 2.2 Diagnostic criteria

The definitions of synchronous multiple primary cancers were based on the criteria of Warren and Gates: (1) each lesion is a pathologically proven malignancy; (2) each lesion must be separated by normal mucosa; and (3) the possibility of metastatic neoplasia should be accurately determined and completely excluded (1). We defined SMPEEC as two or more esophageal neoplasms (high-grade dysplasia [HGD] and early esophageal cancer [EEC]) detected in one endoscopic examination.

In line with the guidelines developed by Warren and Gates, the definitions employed in this study for the main and accessory lesions were established based on the following criteria: (1) Among multiple lesions, those with deeper invasion depth were defined as the main lesions and other lesions were defined as the accessory lesions. (2) If multiple lesions had the same depth of invasion, lesions with longer diameters were defined as the main lesions, and the others were considered as accessory lesions. (3) In cases involving more than two lesions, the second main lesion was defined as an accessory lesion.

Local recurrence was defined as a newly histologically confirmed recurrent cancer at the site where ESD was initially performed, following the initial complete resection. Bleeding related to the procedure was defined as bleeding that required postoperative hemostatic treatment, such as endoscopic clipping or thermocoagulation (8).

### 2.3 Inclusion criteria

All lesions were detected during the endoscopic examination. No treatment was administered before ESD. All lesions were confirmed by histological evaluation of biopsies and classified according to the Japanese classification by the Japan Esophageal Society (9). The inclusion criteria were as follows: (1) confirmation of esophageal lesions as early esophageal cancer or high-grade intraepithelial neoplasia; (2) limited depth of lesions to the mucosa or the submucosa <200µm (3) acceptance with ESD and provision of informed consent.

### 2.4 Exclusion criteria

**Figure 1:**
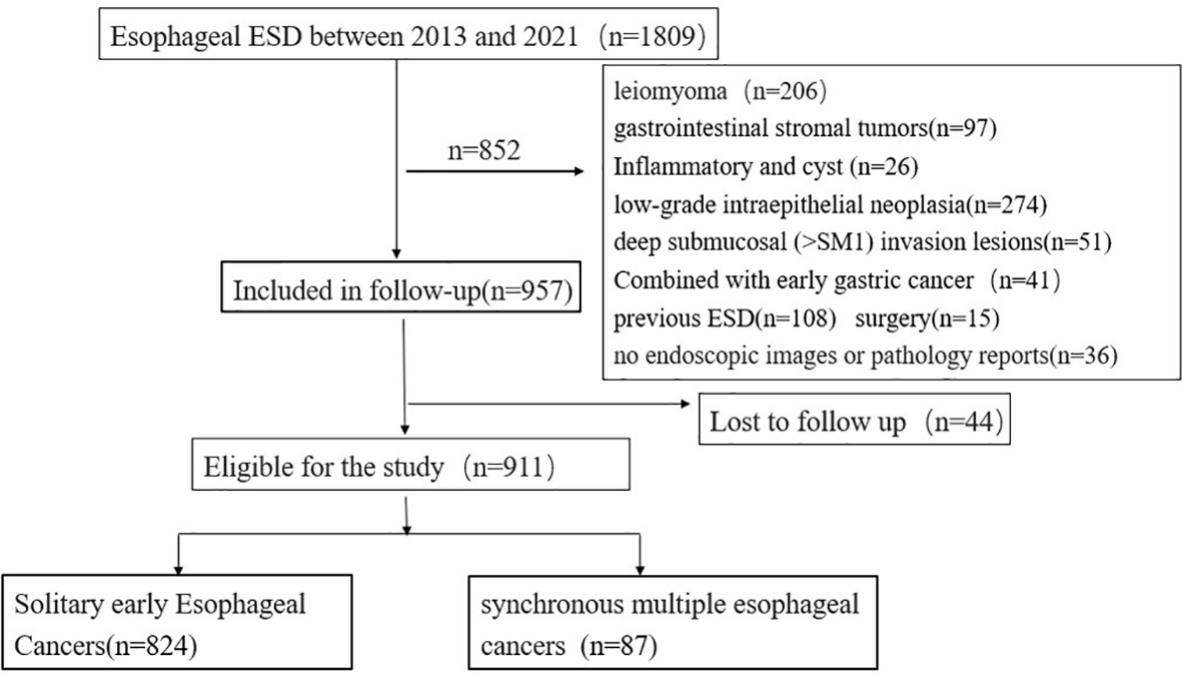
Flow chart of enrolled patients.

Of the 1,809 patients who underwent initial esophageal ESD treatment between 2013 and 2021, 852 were excluded. The exclusion criteria were as follows: (1) esophageal lesions other than early esophageal cancer and high-grade intraepithelial neoplasia: a. low-grade intraepithelial neoplasia (274 patients), b. esophageal leiomyoma (206 patients), c. gastrointestinal stromal tumors (97 patients), d. deep submucosal (SM2) invasion lesions (51 patients), e. inflammatory or cystic lesions (26 patients); (2) patients who underwent ESD (108 patients) or surgery (15 patients) before; and (3) patients without available images of upper endoscopy or pathology reports (36 patients). A total of 44 participants were missing due to unavoidable circumstances (such as loss to follow-up) during the follow-up period (**Figure 1**).

### 2.5 ESD operation

Patients with early esophageal cancer and high-grade intraepithelial neoplasia underwent ESD as the initial treatment. ESD was performed on lesions that met the following criteria: (1) absolute indications: lesions do not exceed the mucosal layer (T1a), which remains within the mucosal epithelium (EP) or the lamina propria mucosae (LPM); (2) relative indications: lesions involving the muscular mucosae or showing slight infiltration into the submucosa (up to 200 μm, T1b-SM1) (10). Esophageal ESD was performed according to the standard technique described previously (11–13). A transparent cap was placed at the front of the endoscope and marked with argon around the left and right walls of the esophagus, and a mixture of saline, methylene blue, and epinephrine was injected into the submucosa of the esophagus. The KD-620LR knife was used to cut the edge of the lesions, and the KD-611L knife was used to gradually separate the lesions. After resection of the lesions, the wounds were treated using KD-610L electrocoagulation and APC hemostasis.

### 2.6 Statistical analyses

Statistical analysis was performed using IBM SPSS version 25.0 (IBM Corp., Armonk, NY, USA) and graphs were generated using GraphPad Prism version 8.0 for Windows (GraphPad Software, La Jolla, California, USA). Categorical variables are presented as proportions, while continuous variables are presented as mean ± SD or median and interquartile range.

Differences between groups were evaluated using the Pearson χ2 test or Fisher’s exact test for categorical variables, as deemed appropriate. Wilcoxon rank-sum tests were used to compare continuous variables. Variables with a *P* value < 0.1 in univariate analysis and variables with major clinical relevance (based on previous studies) were included in the multivariable analysis (binary logistic regression). The cumulative probabilities of multiple lesions were estimated using the Kaplan-Meier method. Odds ratios (OR) and 95% confidence intervals (CIs) were calculated for each variable. A *P* value < 0.05 was regarded as statistically significant.

## 3 Results

### 3.1 Baseline characteristics of patients with esophageal neoplasia

A total of 824 patients with SEEC and 87 patients with SMPEEC were included in this study, and the frequency of synchronous multiple lesions was 9.55% in patients with early esophageal cancer. The average age of the 911 patients enrolled was 64.65± 0.26 years and the male/female ratio was 2.56 (655/256). A comparison of baseline characteristics between solitary and multiple early esophageal cancer patients is summarized in **Table 1**. The proportion of people who consumed alcohol was significantly higher in the group with synchronous multiple neoplasias than in the group without (*P*<0.05). In addition, no significant differences in age, sex, tobacco use, history of chronic disease, or family history of cancer were found between the two groups.

**Table 1:** Baseline characteristics of patients with early esophageal cancer.

	Solitary (%)	Synchronous (%)	*P* value
Number of patients	824	(61,70)	0.242
Gender			0.172
Male	587 (71.2)	68 (78.2)	
Female	237 (28.8)	19 (21.8)	
Smoking			0.069
Yes	288 (35.0)	39 (44.8)	
No	536 (65.0)	48 (55.2)	
Drinking			0.025
Yes	245 (29.7)	36 (41.4)	
No	579 (70.3)	51 (58.6)	
chronic medical history			0.822
Yes	359 (43.6)	39 (44.8)	
No	465 (56.4)	48 (55.2)	
Family history of cancer			0.147
Yes	82 (10.0)	13 (14.9)	
No	742 (90.0)	74 (85.1)	

Solitary, solitary early Esophageal cancer; Synchronous, synchronous multiple primary early esophageal cancer.

### 3.2 Comparison of clinicopathological characteristics of esophageal lesions

The clinicopathological characteristics of esophageal lesions between the two groups are shown in **Table 2**. The median (IQR) diameter of the lesions was 3.5 (2.5–4.3) cm and 2.5(2.0-3.5) cm in the SEEC and SMPEEC groups, respectively, showing no statistical difference. The lesions in the SMPEEC group were mainly located in the lower segment of the esophagus (46[52.9%]), while those in the SEEC group were in the middle segment (412[50.0%]). Most of the macroscopic types in the two groups were flat (80[92.0%] vs. 768[93.2%]). Esophageal cancer appeared in 46.1% and 64.4% of the solitary and multiple groups, respectively. The ratio of esophageal circumference (>1/2 or 3/4) in the SMPEEC group was 31.03%, while the ratio in the SEEC group was 12.38%. There were no differences between the two groups in lymphovascular invasion (12 [1.5%] vs. 3 [3.4%]) and depth of invasion (MM:132[16.0%] vs. 14[16.1%]; SM1:36 [4.4%] vs. 4 [4.6%]). The number of patients who met the absolute indication of ESD was not significantly different between solitary or multiple lesions in the two groups (65 [74.7%] vs. 631 [76.6%], **Table 2**).

**Table 2:** Comparison of clinicopathological characteristics of esophageal lesions.

	Solitary (%)	Main lesion of Synchronous (%)	*P* value
Number of lesions	824	87	
Long diameter (cm), M (P25, P75)	3.5 (2.5,4.3)	2.5 (2.0,3.5)	0.691
Location			0.000
Upper third	68 (8.3)	15 (17.2)	
Middle third	412 (50.0)	26 (29.9)	
Lower third	344 (41.7)	46 (52.9)	
Macroscopic type			0.764
Elevated	34 (4.1)	5 (5.7)	
Flat	768 (93.2)	80 (92.0)	
Depressed	22 (2.7)	2 (2.3)	
Histopathologic type			0.001
HGN	444 (53.9)	31 (35.6)	
Esophageal Cancer	380 (46.1)	56 (64.4)	
Lymphovascular invasion			0.344
Absent	812 (98.5)	84 (96.6)	
Present	12 (1.5)	3 (3.4)	
Depth of invasion			0.974
EP/LPM	656 (79.6)	69 (79.3)	
MM	132 (16.0)	14 (16.1)	
SM1	36 (4.4)	4 (4.6)	
Indications for ESD			0.380
Absolute	631 (76.6)	65 (74.7)	
Relative	181 (22.0)	19 (21.8)	
Lifting sign			0.221
Present	808 (98.1)	83 (95.4)	
Obscure	16 (1.9)	4 (4.6)	
Circumferential rate of lesions			0.0001
<1/2	722 (87.6)	60 (69.0)	
1/2-3/4	41 (5.0)	13 (14.9)	
≥3/4	61 (7.4)	14 (16.1)	

HGD, high-grade dysplasia; EP, epithelium; LPM, lamina propria mucosa; MM, muscularis mucosa; SM1, submucosa<200um.

### 3.3 Treatment outcomes of ESD

ESD is the main therapy for early esophageal cancer; therefore, we compared the effectiveness of ESD treatment between the two groups. As shown in **Table 3**, the median (IQR) operation time was 60 (45-90) minutes in the SEEC group and 90 (60-120) minutes in the SMPEEC group (*P <*0.05). The rate of *en bloc* resection in both groups was higher than 90%. However, the complete resection rate and curative resection rate in multiple lesions was lower compared with solitary lesions (82.8% (72/87) vs. 90.0% (742/824) and 80.5% (70/87) vs. 88.7% (731/824), *P <*0.05).

**Table 3:** Treatment outcomes and complications related to endoscopic submucosal dissection.

	Solitary (%)	Synchronous (%)	*P* value
Number of patients	824	87	
Duration of ESD (min)	60 (45, 90)	90 (60,120)	0.000
En bloc resection	804 (97.6)	82 (93.1)	0.041
Complete resection	742 (90.0)	72 (82.8)	0.036
Curative resection	731 (88.7)	70 (80.5)	0.025
Average number of follow-up months	32 (20,45)	41 (24,65)	<0.001
Adverse events			
Bleeding	36 (4.4)	9 (10.3)	0.029
Micro-perforation	9 (1.1)	1 (1.1)	1
Stricture	130 (15.8)	33 (37.9)	0.000
Local recurrence	15 (1.8)	9 (10.3)	0.000
Additional treatment			
None	775 (93.8)	79 (90.8)	0.356
Yes	51 (6.2)	8 (9.2)	

The median (IQR) follow-up time was 32(20, 45) months for solitary lesions and 41 (26, 45) months for multiple lesions. Bleeding and stricture were more frequently observed in the SMPEEC group than in the SEEC group (4.4% vs. 10.3%, *P*=0.029, 15.8% vs. 37.9%, *P*<0.001). Micro-perforation and additional treatment did not differ between the two groups. However, the local recurrence rate was higher in the SMPEEC group (1.8% vs. 10.3%, *P*=0.000).

### 3.4 Risk factors for multiple esophageal lesions

**Table 4** shows the risk factors for multiple esophageal lesions. On univariate analysis, drinking, pathology, tumor location, and esophageal circumference ratio > 1/2 were found to be independent risk factors for multiple lesions (*P <*0.05). Age, sex, tobacco use, chronic medical history, and family history of cancer were not included. We put factors with a *P* < 0.1 in the logistic regression analysis. Multivariate analysis showed that pathology type (*P*=0.019), tumor location (*P*=0.002), and circumferential rate of lesions (*P* = 0.001) remained independent risk factors for SMPEEC.

**Table 4:** Analysis of risk factors for multiple lesions by logistic-regression analysis.

	B	*P*	O R	95% CI
Drinking	0.435	0.069	1.546	0.966-2.474
Pathology	0.567	0.019	1.763	1.096-2.835
Location		0.002		
Middle	-1.215	0.001	0.297	0.146-0.601
Lower	-0.542	0.112	0.582	0.298-1.134
Cycle		0.001		
1/2-3/4	1.096	0.002	2.993	1.483-6.040
≥3/4	0.950	0.004	2.587	1.344-4.980

Middle, the middle segment of esophagus; Lower, the lower segment of esophagus; Cycle, the ratio of the esophageal circumference.

### 3.5 Characteristics of main and accessory lesions

There were 181 lesions diagnosed histopathologically in 87 patients with synchronous multiple primary early esophageal cancers, and they were simultaneously treated by ESD in a single operation. Eighty Patients (91.95%) had double lesions and seven patients (8.05%) had triple lesions. The morphological and pathological characteristics of the main lesions, which were either histologically more advanced or larger in diameter in cases where the histology was similar, were compared to those of the accessory lesions. The long diameter and sample area of the main lesions were significantly larger than those of the accessory lesions (*P <*0.05). The main lesions primarily exhibited carcinoma as the pathological type (66.7%), whereas the accessory lesions predominantly showed high-grade intraepithelial neoplasia (72.3%). The difference between the main and accessory lesions was statistically significant (*P*=0.000). The shape, location, and indications for ESD did not differ between the main and accessory lesions. The results are presented in **Table 5**.

**Table 5:** Comparison of characteristics between main and accessory lesions.

	Main lesion (%)	Accessory lesion (%)	*P* value
Number of lesions	87	87	
Long diameter (mm), M (P25, P75)	35 (27,43)	25 (20,35)	0.001
Sample size (mm²)	800 (506,1260)	400 (275,700)	0.000
Location			0.179
Upper third	15 (17.2)	15 (17.2)	
Middle third	25 (28.7)	36 (41.4)	
Lower third	47 (54.0)	36 (41.4)	
Macroscopic type			1.000
Elevated	5 (5.7)	4 (4.6)	
Flat	80 (92.0)	81 (93.1)	
Depressed	2 (2.3)	2 (2.3)	
Histopathologic type			0.000*
HGN	29 (33.3)	63 (72.4)	
Esophageal Cancer	58 (66.7)	24 (27.6)	
Depth of invasion			0.010
EP/LPM	70 (80.5)	82 (94.3)	
MM	13 (14.9)	5 (5.7)	
SM1	4 (4.6)	0 (0.0)	
Indications for ESD			0.071
Absolute	72 (82.8)	81 (93.1)	
Relative	13 (14.9)	6 (6.9)	

HGD, high-grade dysplasia; EP, epithelium; LPM, lamina propria mucosa; MM, muscularis mucosa; SM1, submucosa<200um.

**Figure 2:**
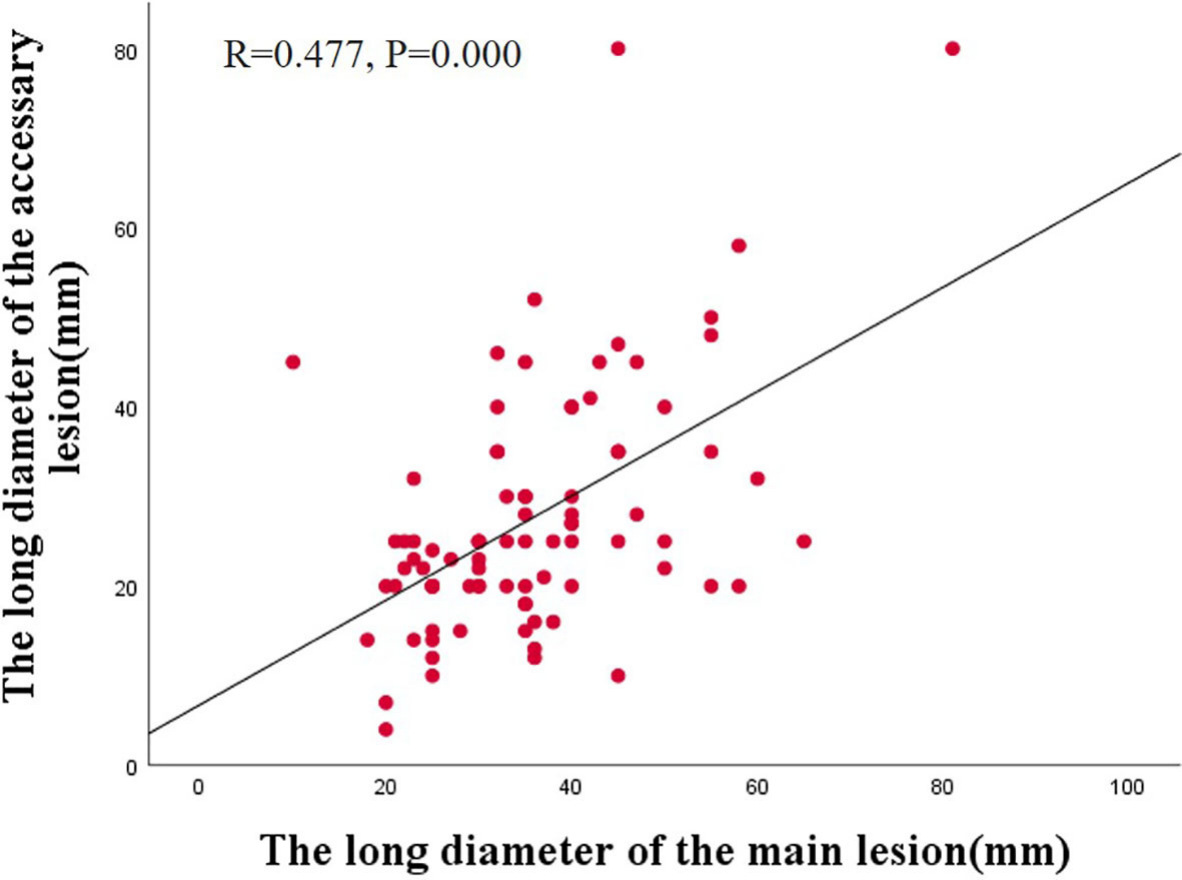
Correlation of tumor diameter between main and accessory lesions of SMPEEC.

Additionally, we observed a correlation between the main and accessory lesions in 87 patients. Spearman’s correlation analysis revealed a positive correlation between the long diameter of the main lesions and the accessory lesions (r =0.477, *P*=0.000) (**Figure 2**). The main and accessory lesions were positively correlated in morphologic type (r=0,632, *P*=0.000), tumor location (r=0.325, *P*=0.037), pathologic type (r=0.299, *P*=0.003), and depth of invasion (r=0.562, *P*=0.000). The results are presented in **Table 6**.

**Table 6:** Comparison of characteristics between main and minor lesions of SMPEEC.

Main lesion	Accessory lesion				r	*P*
**Macroscopic type**	Elevated	Flat	Depressed		0.632	0.000
Elevated	3 (60.0)	2 (40.0)	0 (0.0)			
Flat	1 (1.25)	78 (97.5)	1 (1.25)			
Depressed	0 (0.0)	1 (50.0)	1 (50.0)			
**Location**	Upper third	Middle third	Lower third		0.325	0.037
Upper third	3 (20.0)	9 (60.0)	3 (20.0)			
Middle third	8 (30.8)	9 (34.6)	9 (34.6)			
Lower third	4 (8.7)	17 (37.0)	25 (54.3)			
**Histopathologic type**	HGN	EEC			0.299	0.003
HGN	28 (90.3)	3 (9.7)				
EEC	34 (60.7)	22 (39.3)				
**Invasion Depth**	EP	LPM	MM	SM	0.562	0.000
EP	53 (100.0)	0 (0.0)	0 (0.0)	0 (0.0)		
LPM	12 (75.0)	4 (25.0)	0 (0.0)	0 (0.0)		
MM	10 (71.4)	0 (0.0)	4 (28.6)	0 (0.0)		
SM	4 (100.0)	0 (0.0)	0 (0.0)	0 (0.0)		

HGD, high-grade dysplasia; EEC, early esophageal cancer; EP, epithelium; LPM, lamina propria mucosa; MM, muscularis mucosa; SM1, submucosa<200um.

### 3.6 Long-term outcomes determined by cumulative recurrence rate and stricture

**Figure 3:**
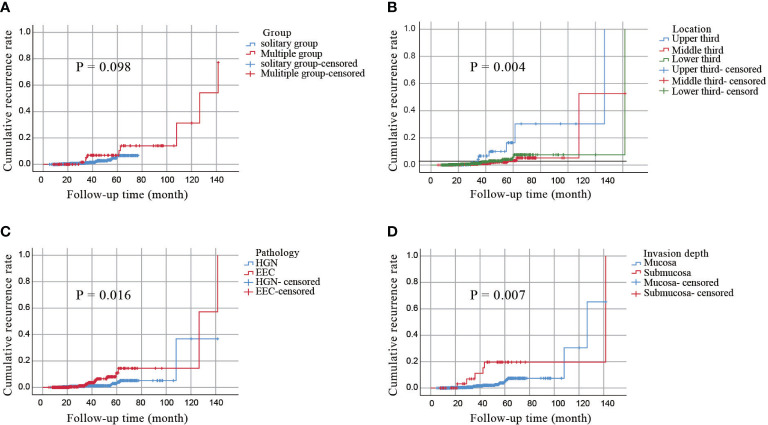
A cumulative incidence of local recurrence by the Kaplan–Meier curves. **(A)** a cumulative incidence of local recurrence in all patients. **(B)** a cumulative incidence of local recurrence of lesions in different locations. **(C)** a cumulative incidence of local recurrence of different pathological types. **(D)** a cumulative incidence of local recurrence with invasion depth.

To further assess the long-term outcomes of ESD for early esophageal cancer, we calculated the cumulative incidence of local recurrence in all subjects (n = 911) using the Kaplan–Meier curves. As shown in **Figure 3A**, the cumulative incidence of local recurrence in multiple lesions was higher than that in solitary lesions, but the difference was not statistically significant (*P*=0.098). However, the cumulative incidence of local recurrence was related to the location of the lesions and was highest in the upper esophagus *(P*=0.004, **Figure 3B**). Additionally, the pathology type and depth of invasion were significantly associated with the cumulative recurrence rate (*P*=0.016, *P*=0.007; **Figures 3C, D**). No significant relationship was observed between the cumulative recurrence rate and lymphovascular invasion, complete resection, or curative resection (**Supplementary Figure 1**).

Subsequently, the potential causes of esophageal stricture in all patients after ESD treatment were investigated. Patients who had multiple lesions, a longer diameter of lesions, and a ratio of the esophageal circumference >1/2 were more likely to have esophageal stricture according to the logistic-regression analysis (P<0.05, **Table 7**).

**Table 7:** Analysis of risk factors for esophageal stricture by logistic-regression analysis.

	B	*P*	OR	95%CI
Cycle		0.000		
1/2-3/4	2.243	0.000	9.422	4.978-17.838
≥3/4	3.690	0.000	40.041	19.26-83.226
Pathology	0.624	0.047	1.867	1.008-3.460
Group	1.009	0.002	2.744	1.458-5.163
Depth		0.947		
LPM	-0.181	0.626	0.835	0.404-1.725
MM	-0.183	0.621	0.833	0.403-1.720
SM1	-0.015	0.979	0.986	0.329-2.957
Long Diameter	0.044	0.000	1.045	1.028-1.062

Cycle, the ratio of the esophageal circumference; LPM, lamina propria mucosa; MM, muscularis mucosa; SM1, submucosa<200um.

## 4 Discussion

Esophageal cancer is the eighth most common type of cancer worldwide and the sixth leading cause of cancer-related deaths. It is characterized by its difficult early diagnosis, high mortality rate, and poor prognosis (14), while surgical treatment is still the first recommended treatment regimen. In recent years, robot-assisted minimally invasive esophagectomy (RAMIE) has achieved better postoperative recovery and reduced complications (15, 16). However, the postoperative survival rate of patients with esophageal cancer remains low (17). Therefore, it is crucial to improve the early diagnosis rate of esophageal cancer, especially for the diagnosis of multiple lesions. SMPEC is a significantly less common condition compared to solitary primary esophageal cancer, with a reported incidence rate ranging between 0.1–10% (3, 4). Furthermore, synchronous multiple primary early esophageal cancer (SMPEEC) is even more infrequent. The prognosis of individuals with multiple esophageal cancers is considerably worse, necessitating treatments such as surgery, radiotherapy, or chemotherapy (5, 18). Very few studies have focused on the treatment of SMPEEC with ESD or the comparison of disease characteristics between SMPEEC and SEEC.

Our study aimed to assess the characteristics and risk factors associated with SMPEEC, while also evaluating the outcomes, recurrence, and stricture rate of ESD in multiple esophageal lesions in comparison to solitary esophageal lesions. The results of our study revealed independent risk factors associated with SMPEEC, along with notable correlations between the main and accessory lesions. The incidence of SMPEEC in this study was 9.55% (87/911), which is consistent with previous reports. No statistically significant differences in age, sex, smoking, history of chronic disease, or family history of cancer were observed between the two groups. However, the proportion of people who drank was significantly higher in SMPEEC than in SEEC (**Table 1**). A possible correlation between alcohol consumption and multiple primary esophageal cancers has been reported by Saeki H and Denggui Wen (19, 20). These authors reported that excessive smoking and alcohol consumption were risk factors associated with multiple primary carcinomas, and the mechanism of the carcinoma manifestation involved increased sensitivity of genes to the environment. These results were consistent with our study.

The en bloc (93.1%) and curative (80.5%) resection rates were satisfactory in the SMPEEC group, similar to previous reports on conventional ESD (21, 22). The predominant complication observed in the SMPEEC group was postoperative stricture, while acceptable levels of bleeding and perforation were observed in both groups. In comparison to the traditional surgical resection of the esophagus, ESD treatment for multiple esophageal cancers provides benefits such as decreased patient pain, reduced costs, and shorter hospital stays. Additionally, ESD treatment offers a higher level of safety and can effectively mitigate complications associated with esophageal cancer (17).

Furthermore, our findings indicated a predominant occurrence of esophageal involvement in the middle segment within the SEEC group, whereas the SMPEEC group exhibited a higher incidence of lesions in the lower segment of the esophagus. Analysis of the histological subtypes in our patient data revealed that esophageal cancer constituted the majority of multiple lesions (64.4%), exhibiting a significantly higher occurrence compared to solitary lesions (46.1%). This indicates that a higher degree of malignancy is more likely to develop SMPEEC. Simultaneously, it was observed that exceeding half of the esophageal circumference significantly increased the risk of developing multiple primary esophageal cancers. Multivariate analysis further proved that the pathology, tumor location, and circumferential rate of lesions were risk factors associated with SMPEEC. When encountering high pathological grade lesions, tumors located in the upper esophagus, and lesions more than half of the esophageal circumference during the endoscopic examination, it is crucial to be vigilant about the possibility of SMPEEC. This study marks the first of its kind in its utilization of regression models for predicting risk factors linked to SMPEEC, offering significant implications for enhancing the diagnostic rate of multiple early esophageal cancers in clinical practice.

Additionally, we analyzed the relationship between the main and accessory lesions in SMPEEC. The main lesion exhibited a significantly larger size compared to the accessory lesion, and a positive association was observed, indicating that as the size of the main lesion increased, so did the size of the accessory lesions. Kim JH reported similar findings in synchronous multiple early gastric cancer (23). There was a positive correlation between the main and accessory lesions in terms of morphologic type, histopathologic type, and invasion depth. Our analysis revealed that the main and accessory lesions shared identical types in 94.3% (82/87) of the cases, while the histopathologic type and invasion depth were consistent in 57.5% (50/87) and 70.1% (61/87) of the cases, respectively. Although there was no notable correlation in the vertical relationship, the main and accessory lesions displayed a considerable consistency rate of 42.5% (37/87). The middle esophagus was the primary site for both the main and accessory lesions, followed by the lower esophagus. These findings lend support to the “field carcinogenesis” hypothesis mentioned in previous studies (24, 25), which provides a plausible explanation for the occurrence of multiple lesions with similar clinicopathological characteristics in the presence of a consistent carcinogenic environment

The cumulative incidence of local recurrence in all included patients was calculated using Kaplan–Meier curves. The 5-year cumulative incidences of the SMPEEC and SEEC groups were 10.46% and 5.73%, respectively. The incidence of SMPEEC was higher than that of SEEC; however, the difference was not statistically significant, which could be attributed to the insufficient follow-up time. With an extended follow-up time, statistically significant differences may be observed between the two groups. A multicenter retrospective study in Japan showed that the local recurrence rate of esophageal mucosal lesions after ESD was approximately 1.9–9.4% (26, 27). Our study provided evidence that the cumulative recurrence rate was affected by tumor location, pathological type, and depth of invasion. The recurrence rate of tumors in our study was the highest in the upper esophagus, which was concurrent with the reports of previous studies (28, 29). The depth of invasion has also been reported to influence recurrence in many studies (30, 31) and our study arrived at the same conclusion. When the depth of invasion reaches the submucosa, the rate of lymph node metastasis increases significantly, which may be the primary reason for postoperative local recurrence.

According to the results of this study, the ratio of esophageal circumference, pathology type, multiple lesions, and diameter length of lesions were risk factors associated with esophageal stricture. It is reported that the incidence of post-ESD stricture in esophageal neoplasms ranges between 5–17% (32–34). A study conducted in Japan reported that postoperative strictures occurred in 90% of patients with lesions exhibiting diameters more than three-fourths of the circumferential extension (35). In our data, the possibility of esophageal stricture increased if a lesion was more than half of the esophageal circumference. The depth of invasion is a known risk factor for esophageal strictures. However, our results revealed no correlation between invasion depth and esophageal stricture. We speculated that this may be due to the inclusion of confounding factors, such as multiple lesions, in our analysis.

There are certain limitations to our study. First, it was performed in a single center and was designed retrospectively. Second, the number of cases in the multiple lesion group was significantly less than that in the single lesion group, so could be a possible chance of bias. Third, some of the included patients did not have enough follow-up time. Therefore, future studies with a larger sample size of patients with multiple lesions and a longer follow-up period would be useful to validate our findings.

Nevertheless, this study has several strengths. This is the first study to comprehensively evaluate the clinicopathological characteristics of primary esophageal neoplasia treated with ESD and identify the risk factors for multiple esophageal lesions. Moreover, we thoroughly investigated the relationship between the main and accessory lesions. Lastly, our findings emphasize the importance for clinicians to be vigilant in identifying potential additional lesions in patients exhibiting these characteristics.

## 5 Conclusion

In conclusion, our study revealed a higher risk of multiple lesions in patients who consumed alcohol. Additionally, our findings indicated that pathology type, tumor location, and circumferential rate of lesions were independent risk factors associated with SMPEEC. We demonstrated that the main and accessory lesions of SMPEEC share similar clinicopathological characteristics. Therefore, when SEEC is detected, it is important not to neglect the possibility of SMPEEC, considering our understanding of the characteristics of the main and accessory lesions.

## Data availability statement

The raw data supporting the conclusions of this article will be made available by the authors, without undue reservation.

## Ethics statement

The studies involving humans were approved by the Institutional Review Board at The First Affiliated Hospital of Nanjing Medical University. The studies were conducted in accordance with the local legislation and institutional requirements. Written informed consent for participation was not required from the participants or the participants’ legal guardians/next of kin in accordance with the national legislation and institutional requirements.

## Author contributions

All authors contributed to the study’s conception and design. JS and JY designed the research; JS, SW, and WL collected the date and wrote the manuscript. JS and HC analyzed the data; LL, LX, and PZ created the tables and figures; JY and GZ reviewed and edited the manuscript. All authors contributed to the article and approved the submitted version.

## Supplementary material

Supplementary Figure 1 A cumulative incidence of local recurrence by the Kaplan–Meier curves. **(A)** a cumulative incidence of local recurrence in different lymphovascular invasion. **(B)** a cumulative incidence of local recurrence of lesions according to complete resection. **(C)** a cumulative incidence of local recurrence according to curative resection.
