# Supplementary figures and images for: Clinicopathological characteristics of synchronous multiple primary early esophageal cancer and risk factors for multiple lesions
Source: Front Oncol. 2023 Aug 17;13:1219451. doi: 10.3389/fonc.2023.1219451 (PMC10471681; doi:10.3389/fonc.2023.1219451)

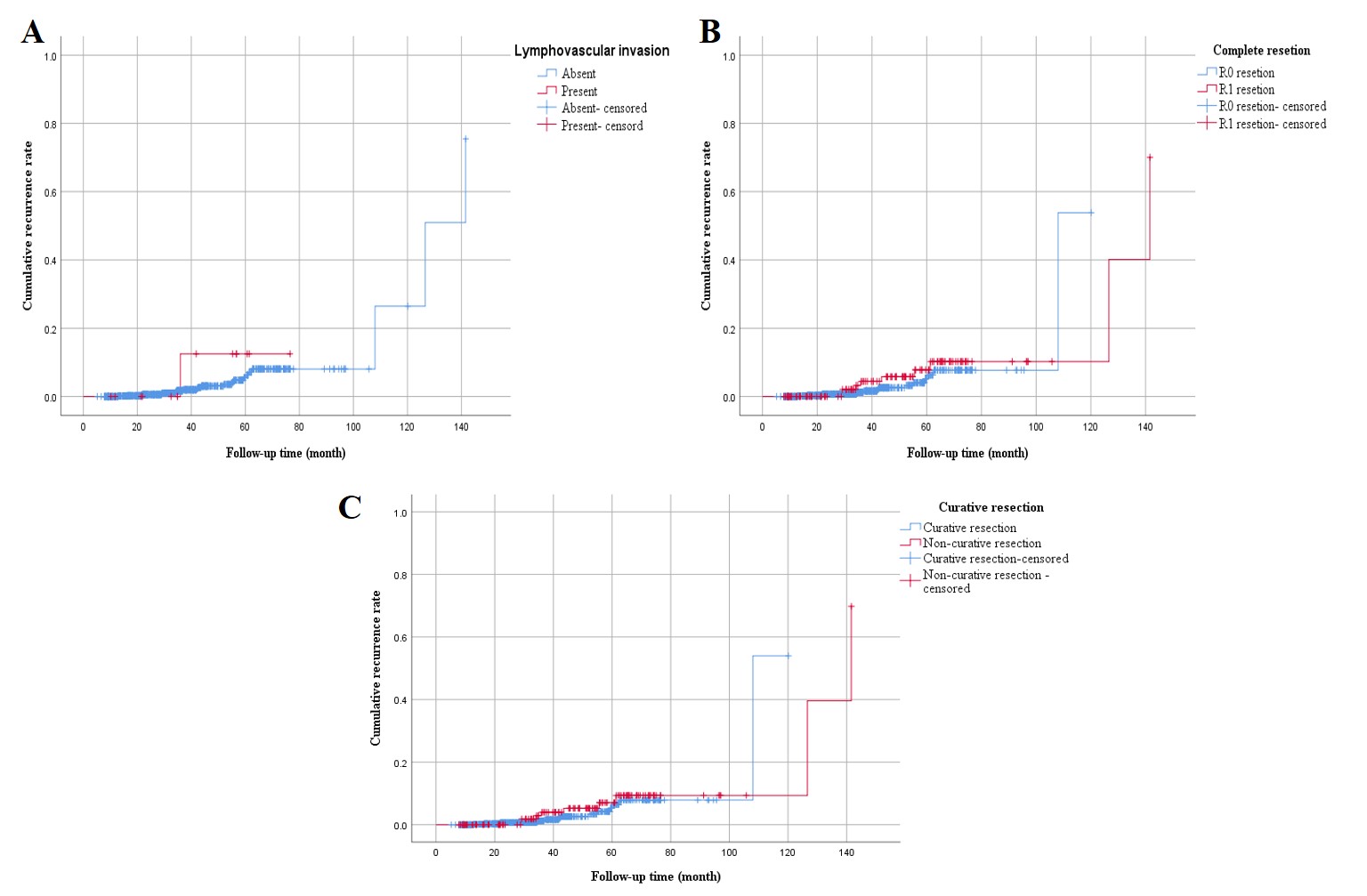

Supplement: Supplementary Figure 1 — A cumulative incidence of local recurrence by the Kaplan–Meier curves. (A) a cumulative incidence of local recurrence in different lymphovascular invasion. (B) a cumulative incidence of local recurrence of lesions according to complete resection. (C) a cumulative incidence of local recurrence according to curative resection. [file Image_1.jpeg]
